# Public Attitudes to Housing Systems for Pregnant Pigs

**DOI:** 10.1371/journal.pone.0141878

**Published:** 2015-11-11

**Authors:** E. B. Ryan, D. Fraser, D. M. Weary

**Affiliations:** Animal Welfare Program, Faculty of Land and Food Systems, University of British Columbia, Vancouver, British Columbia, Canada; Institut Pluridisciplinaire Hubert Curien, FRANCE

## Abstract

Understanding concerns about the welfare of farm animals is important for the development of socially sustainable production practices. This study used an online survey to test how views on group *versus* stall housing for pregnant sows varied when Canadian and US participants were provided information about these systems, including access to scientific papers, YouTube videos, Google images, and a frequently-asked-questions page (S1 Appendix). Initial responses and changes in responses after accessing the information were analyzed from Likert scores of 242 participants and from their written comments. Participants were less willing to accept the use of gestation stalls after viewing information on sow housing. For example, initially 30.4% of respondents indicated that they supported the use of gestation stalls; this declined to 17.8% after participants were provided additional information. Qualitative analysis of comments showed that supporters of gestation stalls expressed concern about the spread of disease and aggression between animals in less confined systems, whereas supporters of group housing placed more emphasis on the sow’s ability to interact socially and perform natural behaviors. These results point to public opposition to the use of gestation stalls, and indicate that the more that the public learns about gestation stalls the less willing they will be to accept their use.

## Introduction

Pregnant sows are often housed singly in gestation stalls [1]. Gestation stalls enable individual monitoring and feeding by the producer but allow the animals only minimal physical movement. One alternative is group pens that allow greater freedom of movement but can also expose sows to aggression from pen-mates [2]. Citizens are showing increasing interest in how farm animals are reared [3,4], and the use of gestation stalls has become one focus of public attention [5]. Because the social sustainability of animal production practices requires public acceptance, some means of assessing these attitudes is needed for effective policy.

Much of the research to date has focused on the public as consumers [5], including willingness to pay for products associated with higher welfare standards. Less work has focused on the public as citizens who can use political processes to express their ethical concerns [6]. Research on attitudes towards pig production methods has also focused predominantly on Europeans; little is known about the attitudes of North Americans on the issue of sow housing.

One common criticism of research on public attitudes to farming practices is that individuals lack awareness of the real-life conditions on farms and that their responses therefore reflect misconceptions. Hence, there is a need to understand how the public responds to information when this is provided. The aim of this study therefore was to 1) provide quantitative tests of the effect of providing additional information about gestation stall and group housing systems, and the effects of participant demographics, on the attitudes of participants and 2) provide qualitative descriptions of the reasons why some participants changed their responses after viewing the additional information, and more generally their reasons for supporting different methods of sow housing. Because expert views on sow welfare generally identify advantages and disadvantages with both housing systems rather than simple opposition to stalls as promoted by critics, we predicted that participants would become more supportive of the use of gestation stalls after exposure to additional information.

## Methods

This study used the University of British Columbia’s “Your Views” platform (www.yourviews.ubc.ca), following the methodology of Ventura et al. [7], Weary et al. [8] and Schuppli et al. [9]. YourViews provides an interactive, on-line forum to allow participants to engage with the views of others on ethically challenging issues in technology and science [10]. This study, including the method used to gather consent, was approved by the Behavioral Research Ethics Board of the University of British Columbia.

### Survey design

All YourViews surveys begin with demographic questions on age, gender, level of education, and country of residence. Respondents provided responses to two questions (for later quantitative analysis) and qualitative comments describing their rationale [11]. Participants were assigned randomly generated pseudonyms that were used to identity reasons they provided.

When first entering the survey, participants were required to give their consent to participate in the study by clicking on “I consent to participating in this study” before continuing. Consenting participants were then presented with the following synopsis of the sow housing debate, and the perceived benefits and drawbacks of gestation stalls and group housing (Table 1):

**Table 1:** The perceived benefits and drawbacks of gestation stalls and group housing systems that were presented to participants upon entering the survey.

	Gestation Stalls	Group Housing
**Proponents suggest that**	Limit aggression	Increases social contact between animals
	Limit the spread of disease between animals	Increases animals’ ability to move
	Make pork production more efficient	Improves bone, muscle, and joint health
	Allow for animals to be fed individually	Improves animals’ ability to perform natural behaviors
**Opponents suggest that**	Limit animals’ ability to move	Increases aggression between animals
	Limit animals’ ability to socially interact	Results in wounds that affect animal welfare
	Limit animals’ ability to perform natural behaviors	Can affect workers’ ability to monitor animals individually

In an effort to reduce production costs and to simplify the management of animals, intensive housing for pregnant sows and gilts began to increase in the 1950’s. Throughout most of the industrialized world, pregnant sows and gilts are kept in confined housing, known as gestation stalls or gestation crates, for most of their four-month pregnancy. However, some U.S. states and the European Union have banned the practice, calling for animals to be housed in groups. Outlined below are the perceived benefits and drawbacks of gestation stalls and group housing.

Respondents were then asked, “Do you believe that pregnant sows should be housed in gestation stalls or in groups? Please explain”. Decision categories were: “I strongly believe sows should be housed in gestation stalls because…”, “I moderately believe sows should be housed in gestation stalls because…”, “I am neutral on this issue because…”, “I moderately believe sows should be housed in groups because…”, “I strongly believe sows should be housed in groups because…”, and “I don’t believe sows should be housed in either gestation stalls or groups because…”. After selecting a decision category participants could add a comment explaining their response, or ‘vote’ for one or more of the comments left by previous participants.

On entering the survey, participants were assigned to group. The comments given by participants were added to a list on the screen that was visible only to participants in the same group; future participants in the same group were able to choose from the list of reasons already given as well as add their own. The order of listing reasons was determined by an algorithm that placed more recent and more popular reasons (i.e., reasons with the most votes) at the top [11]. Thus later participants within a group were exposed to a larger number of reasons than earlier participants. By allowing participants to see the responses of other group members, new responses were formed in a context analogous to online forums where consensus can develop from the ongoing discussion. Participants were able to vote for as many reasons as they wished but multiple selections were discounted proportionately (e.g., voting for 3 reasons resulted in each reason receiving 0.33 votes).

After participants had responded to the initial question they were able to view additional information including scientific review papers on sow housing by Gonyou [12] and Rhodes et al. [2], a frequently-asked-questions page (S1 Appendix) that was compiled by the first author in consultation with specialists in animal welfare and sow housing, and Google images and YouTube videos obtained using the search terms “Sow gestation stalls” (see live link to images and videos) and “Pregnant sow group housing” (see live link to images and videos). Our intention was to mimic the real-world actions of concerned citizens; we therefore provided convenient access to material but allowed participants free choice of what information they would access. We later asked participants to indicate which information they had viewed. All information was provided in links that opened in new windows. The Google and YouTube images displayed changed over time according to the proprietary algorithms; images included stalls with injured animals posted by advocacy groups, industry-sponsored imagery of clean gestation stalls, and images of group housing with slatted (usually concrete) floors or straw.

To determine if participant responses changed after the provision of information participants were asked, “Given the information you have now accessed, has your attitude changed? Do you believe that pregnant sows should be housed in gestation stalls or in groups? Please explain.” The same decision categories described above were again available. Previous reasons were listed in the same order as described above.

### Participant recruitment

Participants were recruited in three phases between May 9, 2012 and September 25, 2012. The first phase (Group 1) was intended to pilot the survey and elicit a range of responses from concerned participants; these participants (n = 87) were recruited using purposive sampling [13] by advertising with posters (on community boards within the City of Vancouver and at the Vancouver Farmers’ Market), on websites (including that of the British Columbia Society for the Prevention of Cruelty to Animals and UBC’s Faculty of Land and Food Systems), and via email sent by Canada’s National Farm Animal Care Council (NFACC). The survey was also distributed to individuals on Facebook using “snowball” sampling [14]. These participants were predominantly female (77.0%), Canadian (96.4%), educated (94.3% with at least a college level of education), and young (29.4% were between 19 and 29; 30.4% were between 30 and 39 years old).

The second phase was intended to test the effect of treatment and demographic variables using groups from Canada (Groups 2 to 5, hereafter referred to as Canada M-Turk; n = 58) and the US (Groups 6 to 9, hereafter referred to as US M-Turk; n = 77) recruited using a commercially available recruitment tool (Amazon’s crowd-sourcing service, Mechanical-Turk). Mechanical-Turk is known to provide access to respondents who are more attentive and more demographically diverse than standard Internet samples or samples of college students [15]; therefore, the M-Turk participants (n = 135) were also used to analyze the effects of participant demographics on responses. The M-Turk participants were predominantly female (60.1%), educated (77.7% at least college), and young (54.9% were between 19 and 29; 20.3% were between 30 and 39 years old).

The final phase (Group 10; n = 20) was intended to elicit comments from a sample of individuals who were highly engaged and informed on issues regarding farm animal welfare. These participants were recruited from the 3rd-year undergraduate Animals and Society course at UBC. These participants were predominantly female (75.0%); by design all were Canadian residents, college educated, and predominantly young (90% were between 19 and 29 years old).

Group 1 contained the most participants, as this initial group was used to assess the effect of group size in eliciting qualitative comments from the respondents. Preliminary analysis of Group 1 showed that the rate at which new reasons were added declined as more participants entered a group and votes tended to coalesce around a few prominent reasons. Based on this experience and other research using this platform [16], new groups were started after approximately 20 (range 12 to 26) participants had contributed.

### Analyses

We used both quantitative and qualitative methods to achieve our aims. Quantitative analysis was restricted to Mechanical Turk participant responses, which allowed us to test the effect providing additional information, and the effects of participant demographics, on the attitudes of participants (see detailed description below). Qualitative analysis of comments was used to describe the reasons why some participants changed their responses after viewing the additional information, and their reasons for supporting different methods of sow housing.

#### Quantitative analysis

Quantitative analysis was restricted to the groups recruited via Mechanical Turk (Groups 2–9). All data were analyzed using SAS statistical software (Version 9.3) (data deposited in the UBC Research Data Collection dataverse repository http://hdl.handle.net/11272/10219). The N-Reasons platform allowed participants to vote for as many reasons as they wished in each question. In 15 cases this resulted in participants selecting reasons linked to different Likert-scale ratings, for example selecting one reason for moderate support of groups and another reason for strong support; these participants were removed from the analysis. This resulted in a total sample of 135 participants who responded both before and after the provision of the additional information.

Each participant provided an independent test of the prediction that support for stalls would change when participants were provided more information about sow housing methods. Thus changes in participant responses from before to after the provision of the additional information were tested using a within-subject Friedman’s test (n = 135).

Demographic categories were as selected by the respondents: gender (female, male), age (19 to 29, 30 to 39, 40 to 49 and 50 years old or higher) country of residence (Canada, US), and level of education (secondary, college/university and post graduate). Not all respondents provided demographic responses to all questions, so sample size varied from 127 to 135. The first step was to examine univariable associations between demographic variables and support for gestation stall use (i.e. combined strong and moderate supporters of stalls versus others) using Chi-square tests. Demographic variables found to be significant (P<0.05) in the univariable analysis were included in the multivariable test. A mixed model logistic regression (GLIMMIX in SAS) was used to test how support for stalls (*versus* other responses) varied with demographics. The model specified a binary response distribution, a logit link and group as a random effect.

#### Qualitative analysis

Qualitative analysis of comments provided by participants was used to describe 1) the reasons why some participants changed their responses after viewing the additional information, and more generally, 2) their reasons for supporting different methods of sow housing. For the former, we analyzed 40 pairs of comments (i.e., responses to the first and second question) from respondents who changed their views after accessing the additional information, or who specifically commented on the effect of the additional information. For the latter, we analyzed all 99 comments provided by the participants in response to the first question.

Qualitative content analysis (within the framework of qualitative description) [17] guided the coding process used by the primary author (E. Ryan) and a research assistant (N. Bobadilla) to interpret participant comments. Both coders began by independently reading comments within each question, multiple times, with an emphasis on identifying common issues that participants expressed [17]. Brief codes (e.g., management challenges, social opportunities, aggression), were used to identify the key issues expressed by participants. The two coders met to compare their coding and resolved any inconsistencies. Once a high level of consistency between coders had been achieved, the primary author then organized codes under themes relating to issues associated with sow housing (e.g., physical health, movement)[18]. Within the set of comments, the number of times that themes were referenced was counted. A theme was only counted once within each comment, regardless of how many times it was referenced within that comment. Following Ventura et al. [7] we focused on how themes were used to explain support for the different housing systems.

In the Results section we provide quotes that illustrate the issues of concern, relative to themes. We report the group that the comment originated from (described in Participant Recruitment) and the votes associated with the comment. For example, a notation of (2; [1]) describes a comment from group 2 that received a total of one vote. We report both common and less frequently articulated themes and comments (i.e. those that have many and few votes) to reflect the diversity of views [18,19].

## Results

### Quantitative analysis

**Fig 1:**
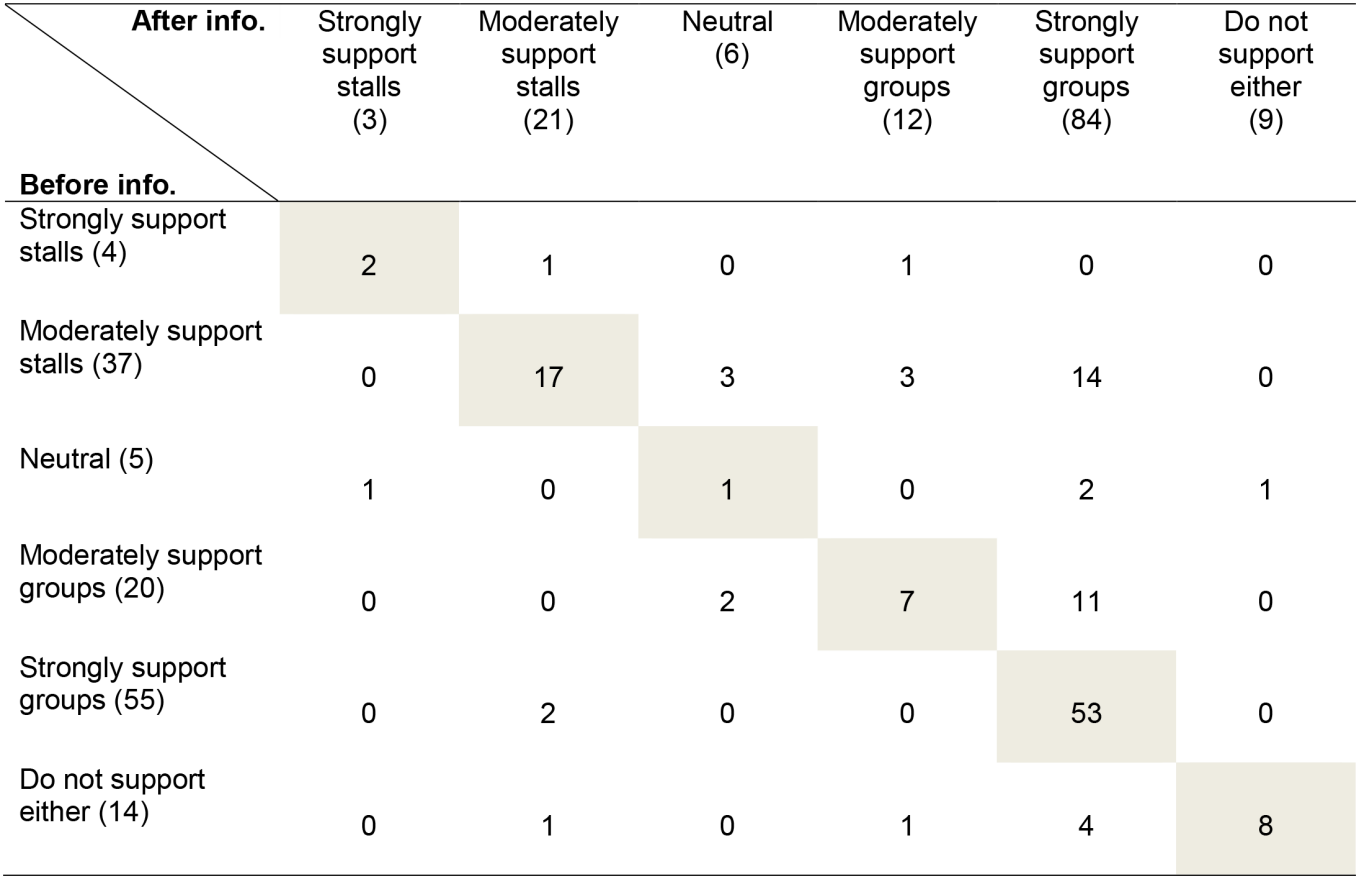
Responses to the question “Do you believe that pregnant sows should be housed in gestation stalls or in groups?” Responses were provided before the provision of additional information and after participants had the opportunity to view images, videos, scientific review papers, and a frequently-asked-questions page. Each cell shows the number of participants (out of a total of 135 respondents recruited via Mechanical Turk) adopting each pair of responses. The number in parentheses in the left-hand column shows the before totals and the number in parentheses in the column headings shows the totals after respondents had viewed the additional information.

Considering only the Mechanical-Turk participants (Fig 1), prior to the provision of additional information, 55.6% supported group housing (moderate and strong support), 30.4% supported gestation stalls (moderate and strong support), 3.7% of respondents were neutral, and 10.4% did not support either system. Contrary to our prediction, the provision of additional information generally reduced support for the use of gestation stalls (Fig 1; P<0.001). In particular, more people strongly supported group housing after accessing information (84/135) than before (55/135). Of the 88 participants who maintained their position after the provision of additional information, most (53) were the strong supporters of group housing.

Respondents were able to consider as many different sources of additional information as they desired. The majority of respondents reported having looked at images or videos of either gestation stalls or group housing for pregnant sows (121/135). Thirty-two percent said they looked at one or both scientific papers; the same number also reported reading the frequently-asked-questions page.

One strength of our methodology was the use of independent groups. Each of the groups was exposed to a different set of participant comments, allowing us to assess the generality of our results. In each of 8 replicate groups recruited using Mechanical Turk, most participants did not support gestation stalls before the provision of the additional information (support ranged from 0.0 to 44.5% support for stalls; moderate and strong support combined, see Table 2). After the provision of information, support declined in five of the groups, stayed the same in two groups and increased (by one participant) in one group.

**Table 2:** Participant support for gestation stall housing (moderate or strong support for gestation stalls) before and after access to addition information. Results are shown in relation to participant demographics. The participants shown here were recruited via Mechanical-Turk and provided demographic information (n = 133). Participants responded to the question “Do you believe that pregnant sows should be housed in gestation stalls or in groups?”.

		Support for gestation stalls (%)	
Demographic		Before access to information	After access to information
*Gender*			
	Female (n = 80)	27.5	10.0
	Male (n = 53)	34.0	28.3
*Age*			
	19–29 (n = 73)	37.0	17.8
	30–39 (n = 27)	33.3	22.2
	40–49 (n = 18)	22.2	22.2
	50+ (n = 15)	6.7	6.7
*Education*			
	Secondary (n = 29)	51.7	34.5
	College (n = 88)	23.9	12.5
	Post graduate (n = 13)	30.8	15.4
*Country of Residence*			
	Canada (n = 58)	20.0	9.1
	U.S.A. (n = 77)	37.5	23.8
*Group*			
	2 (n = 13)	0.0	0.0
	3 (n = 12)	41.7	50.0
	4 (n = 15)	20.0	0.0
	5 (n = 18)	22.2	0.0
	6 (n = 16)	12.5	12.5
	7 (n = 22)	44.5	36.4
	8 (n = 18)	44.4	22.2
	9 (n = 21)	42.9	19.1

The decline in support after additional information was also consistent across most demographic categories, specifically across both males and females, both U.S. and Canadian residents, and for all three levels of education (Table 2). Support also declined after the provision of information for the younger participants (19 to 29 and 30 to 39); there was no decline for the older participants, but support for stalls was already low for these individuals.

Support for stalls varied across the demographic categories. Before the provision of additional information, univariable analysis showed that U.S. residents were more supportive of stalls than were Canadians (P<0.03) and participants with less education were more supportive of stalls (P<0.02). In the multivariable analysis only education was significant (P<0.04). After the provision of information, univariable analysis showed similar effects of participant residency (P<0.03) and education (P<0.03), and also showed that males were more supportive of stalls than were females (P<0.01); the multivariable analysis also showed effects of gender (P<0.04) and education (P<0.05).

### Qualitative analysis

The comments provided by participants provided insights into why they changed their response after viewing the information. Below we describe these comments in relation to the participant’s original response.

The majority of participants who were moderate in their support of either system, or who were neutral on the issue, changed after accessing sources. The following comment is from one participant who was initially a moderate supporter of group housing and who became a strong supporter of groups: *“…after seeing photos of the stalls…I believe they should be housed in groups because it’s the best of two bad choices*.*”* (8; [0.7])

A moderate supporter of gestation stalls initially justified stall housing with this comment:*“It seems that this method has been working since the 1950s*, *and I believe it is important to avoid the spread of diseases above all*, *especially if the pigs are used for meat*.*”* (4; [3.25]) After accessing addition information the same respondent wrote: *“I did not realize that the stalls were so small*. *Knowing the size of the stalls*, *I think it is completely unfair and unreasonable to leave a pregnant pig unable to move for part or all of the pregnancy*.*”* [10.5]

Two participants who strongly supported groups before accessing additional information became moderate supporters of gestation stalls. One of these participants remarked that, *“The sight of the wounds caused due to grouping makes me change my reason*.*”* (6; [1]) One participant who strongly supported gestation stalls before the provision of information became more moderate in their support of gestation stalls, and another switched to moderate support for group housing. The latter of these two participants described their reason for switching: *“I thought that the gestation stalls would be cleaner and have more room but they were tiny and they said the sows stayed in there for the duration of their lives*, *so it's probably better for them to be housed in groups*.*”* (8; [1.2])

Nine of the comments relating to change were made by respondents who did not actually change their decision. Of these, 7 were from strong supporters of group housing, 1 from an individual who did not support either system, and 1 from a moderate supporter of stalls. These participants claimed that the information either had no effect or that it intensified their position. For example, one strong supporter of group housing commented: *“Viewing the images of sows in gestation stalls renewed and strengthened my opposition to them*.*”* (2; [1])

The comments provided by participants also provided insights into their reasons for supporting different methods of sow housing. Many of the comments focused on one or more of three types of concerns: physical health (e.g., disease and injury), the ability to live in a “natural” manner (e.g., ability to perform natural behaviors and live in environments with natural elements), and affective states (e.g., pleasure and pain) (Table 3). We describe the themes raised by participants that supported gestation stalls, and then describe themes raised by supporters of group housing.

**Table 3:** Comments (n = 99) provided by all participants in response to the first question (before the provision of additional information) were coded according to the themes referenced.

Theme^1^	Description	Comments^2^	Votes^3^	Expressed by^4^
**Physical health and well-being**	Comments regarding health, feed intake, disease, mortality, including potentially injurious aggression and means of controlling it	44	113.5	All
**Social interaction**	Comments regarding access to other pigs and social isolation	41	175.4	All
**Movement**	Comments regarding space allowance and exercise	21	102.5	All
**Natural behavior**	Comments regarding rooting, nest building, access to straw, and ability for to perform motivated behaviors	19	105.7	Not strong support stalls
**Affective states**	Comments regarding sentience, stress, happiness, pain, suffering, mental health, and psychological distress	15	30.3	All but strong stall supporters
**Natural environments and evolution of behavior**	Comments about natural environments and how animals have evolved	13	50.6	Not neutral or strong support stalls

^1^ Themes were only counted once per comment no matter how often the theme was referenced. Each comment could reference multiple themes.

^2^ Denotes the number of comments that referenced the theme.

^3^ Votes associated with each comment that referenced the theme. Participants could vote for multiple comments in which case these were discounted (e.g. if two reasons were selected by a single participant, each was assigned 0.5 votes).

^4^ Decision groups that expressed comments included strong and moderate supporters of gestation stalls and group housing, those that were neutral on the issue, and those that chose not to support either system.

Below we describe each theme and the number of comments within decision category (e.g., strong or moderate support of either system) that referenced these. Moderate and strong supporters of stalls tended to highlight concern for physical health, including spread of disease (7/19 comments), the importance of individual monitoring of sows (5 comments), and issues associated with injuries and aggression (11 comments). For example, one strong supporter of gestation stalls said:

*“I grew up on a farm where we originally had sows housed in groups and they were very aggressive toward each other*. *We later changed to gestation stalls and both the sows and their offspring were in better health and were less stressed than they had been in groups*.*”* (8; [1.5])

Concern for the animals’ safety and ease of labor were voiced by a moderate supporter of gestation stalls: *“I think the lowering of aggression*, *and the fact that the workers can individually monitor [the sows] sounds nice*.*”* (7; [9])

Moderate supporters of stalls tended to prioritize protection from injury or disease, although one moderate stall supporter acknowledged that there may be a need to provide for social interaction:

*“They are being raised for food*, *which means that curbing disease and injury is in the best interest of those involved; however*, *if they are to be bred repeatedly*, *it raises the question of what manner of social structure pigs form naturally*. *If it would be beneficial for them to be kept in a group of other animals…it should be considered*.*”* (3; [4])

Some gestation stall supporters described the need for the management of animals and their environments. A moderate supporter of stalls suggested that domestication may have reduced how much sows benefit from social interactions:

*“They [sows] evolved to live in social packs out in the wild [but] have been bred …away from that*, *and their lives are nowhere close to what they are in the wild*. *Having them congregate while pregnant at the cost of injuries and disease is a token gesture that does nothing to actually help the pig and does much to hurt the farmers*.*”* (6; [1])

Concern for the affective states of sows was mentioned in just two comments from stall supporters. For example, one moderate supporter of gestation stalls said that stalls were, *“ok only if…the sow does not show any signs of pain*.*”* (9; [3])

Supporters of group housing (moderate and strong support) favored the ability of pigs to interact socially, even if this occasionally resulted in some competitive or aggressive behavior. Thus when these participants mentioned aggression (14/58 comments) they mainly focused on how management of groups could prevent the problem. These comments focused on stocking density, group composition, and approaches to feeding that reduce aggression. For example, one respondent who strongly supported groups suggested:

*“The only reason sows are aggressive in group housing is because the [sows] …are crowded in small enclosures where they feel the need to be territorial*. *I’ve raised pigs and as long as they have adequate space and things to do (root*, *nest*, *etc*.*) they are very rarely aggressive enough to cause injury*.*”* (5; [5])

Another questioned the assertion by some supporters of gestation stalls that group housing puts animals at risk for aggressive interactions:

*“…Why does group housing risk aggressive behavior*? *Perhaps the group housing situations are too constrictive*? *Or could it be that pregnant sows need ‘areas of refuge’*? *Maybe we should look at what group housing looks [like] instead of assuming that gestation [stalls] are the only alternative*.*”* (2; [1])

Moderate and strong supporters of groups often commented on the benefits of social interaction (28/58 comments), space for greater movement (13 comments), expression of natural behavior (14 comments—including 5 comments on rooting and nesting behaviors). For example, one respondent commented:

*“Pigs are social animals and I think they should have the opportunity to engage in social interactions with other pigs or decide [when] they want to be alone*. *As well*, *pigs need an area to walk about and have the opportunity to perform behaviors they are motivated to do*.*”* (1; [6.6])

Five comments focused on the idea of natural environments and the pigs’ ‘nature’, with the majority of comments reflecting the belief that a good life for animals could be achieved by minimizing human interference or emulating nature. Moderate and strong supporters of groups used language that expressed a desire to see sows *“live naturally”* and referred to living conditions of wild pigs to support their assertion that sows’ adaptive behaviors are thwarted in certain captive settings. One strong supporter of group housing situated the issue of behavioral deprivation in the context of evolution:

*“Ethological studies have proven again and again that removing animals from their natural conditions causes stress*. *Pigs have evolved for thousands of years to be herd animals…To confine a sow*, *apart from the direct*, *physiological cruelty inherent in such a situation is [to force] her into a situation which goes against all of her natural instincts (namely to root and forage with a herd)*.*”* (2; [7])

The comment above, while focused on living naturally, also references concern for sows’ mental and emotional states. Nine other comments referred to the sows’ capacity to feel stress, pain, and happiness.

## Discussion

Contrary to our prediction that more information provided would result in greater support for stalls, our results showed that the majority of participants preferred pregnant sows to be housed in groups (56%), especially after accessing additional information on the issue (71%). Earlier work on Flemish participants also linked good animal welfare with elements of social housing[20]. The use of gestation stalls, when perceived negatively by the public, may result in distrust of industries and their products [21,22] and increased support for legislative change. The support for group housing in this study is consistent with public sentiment reflected in ballot initiatives in various states of the US calling for bans on gestation stalls [23].

Participants became more supportive of group housing after accessing information. Ninety percent of participants viewed images or videos. Participants commented on the capacity of visual information to change their views. Research on visual methods, including graphics and animations, has shown the impact these images can have [24]. Photographs or video may be perceived as particularly useful by participants who are removed from agricultural practices. Internet images and videos are increasingly used to inform perceptions of the world [6].

Access to multiple sources of information has transformed how many individuals manage information related to other issues including human health [25]. In addition, Frewer and Salter demonstrate that as a result of increased levels of education and access to information, people are now less reliant on expert advice to inform their opinions on societal events [21].

One limitation of our research was that we were unable to track what images participants looked at. We suggest that future research look into which types of visual images are effective in changing participants’ positions on ethical issues. Further work is also required on participant perceptions regarding the accuracy of different sources in depicting the situation. We suggest that future studies test the effect of access to specific information, for example, by providing participants with only Internet images versus provision of scientific information on the issue.

The majority of participants (more than 60%) did not change their response after exposure to additional information. Previous research has shown that once people have stated a view they tend to favor information that supports maintenance of that view [26]. The design of our study, which asked people to state their view before and after the provision of information, may have underestimated the effect of information, as at least some participants may have been reluctant to change their stance. Future studies should consider using between-subject designs, with participants randomly assigned to different information treatments.

Some support for gestation stalls was rooted in concerns regarding pig health, with participants suggesting that the spread of disease is reduced in gestation stalls. There is still a lack of consensus in the scientific literature regarding the effects of housing system on the spread of disease. For example, Rhodes et al. [2] reviewed scientific literature related to housing systems and health and concluded that health is affected by many factors unrelated to housing methods, including “daily management, pathogen exposure, geographic location, and biosecurity measures” (p. 1585). Concerns regarding health may have been primed by the background information we provided to participants on the “perceived benefits” of each system. Specifically, we noted that one perceived benefit of housing sows in gestation stalls was that it “may limit the spread of disease between animals”.

Group supporters used language that expressed a desire to see sows “live naturally” and referred to living conditions of wild pigs to support their assertion that the sows’ natural behaviors are thwarted in certain captive settings. The idea that a good life for animals could be achieved either by minimizing human interference or emulating nature was also noted by Boogaard et al. [27]. Research on European attitudes towards pig rearing methods has revealed high levels of support for systems that are perceived to be more natural [28]. With respect to providing environments that allow for natural behavior, some comments from supporters of groups mentioned the need for straw or substrates that enable rooting; these participants seemed to be unaware that group housing systems do not necessarily provide straw or similar substrates [29]. It is possible our participants accessed additional information that showed group housing with straw. Other work has shown that perceptions of how well pig housing systems meet the animal’s needs are affected by the availability of straw or other substrate [30]. Further research would benefit from exploring attitudes when straw is provided with and without grouping.

Many of the supporters of groups mentioned the benefits of social contact, but only two studies to date [31,32] have scientifically assessed the motivation of sows to access a group pen. This research has shown that sows are weakly motivated to access a group pen with slatted flooring [33] but more strongly motivated to do so when the group pen is enriched (i.e., with material including straw, compost, rubber matting, and cotton ropes) [32]. Future research on social behavior in pigs living in commercial systems is needed to understand the aspects of socialization that pigs find to be important.

For both stall and group-housing supporters, there was a shared concern regarding aggression in groups. The comments of stall supporters suggested that this housing method decreased the labor necessary to manage animals and aggression associated with grouping; previous work has shown that Dutch citizens also valued innovations that reduced the amount of labor required in farming [27]. Group-housing supporters typically argued that any aggression was due to how groups were managed, and that with the right management benefits of natural social behaviors would outweigh any problems with aggression. Participants advocated for the use of feeding systems, stocking densities, group composition, etc., that reduced the risk of aggressive interactions. Previous research on European participants has shown that many believe that farmers have the responsibility to manage herds in such a way as to address issues of public concern [34]. Other work has highlighted the importance of allowing natural behaviors as well as the concern that animals may be placed in crowded pens that increase the risk of aggression and injury [3,35]. Some supporters of both housing systems appeared to recognize the complexity of choosing one system over the other and hesitated to specify ‘one-size-fits-all’ solutions to sow housing [23].

Participants in all decision groups, except for strong supporters of gestation stalls, expressed concern about sows’ mental states. Concerns related to eliminating negative affective states such as pain, and promoting positive affective states, such pleasure associated with social interactions and natural behaviors. Positive affective states are increasingly viewed as important in the animal welfare literature [36] underscoring the need for housing that allows for positive aspects of welfare and not only the absence of negative affective states.

## Conclusions

Participants supported group housing for sows and became more supportive after exposure to additional information about the different housing systems. Qualitative analysis revealed the importance that participants placed on the provision of social interaction and environments that allow pigs to express natural behavior. Together these results suggest the widespread use of gestation stalls in the pig production is unsustainable.

## Supporting Information

S1 Appendix Frequently-asked-questions page. Common questions and answers around the issue of housing pregnant pigs, provided to all participants, as a source of additional information via a hyperlink.
(PDF)
