# Supplementary material for: Public Attitudes to Housing Systems for Pregnant Pigs
Source: PLoS One. 2015 Nov 11;10(11):e0141878. doi: 10.1371/journal.pone.0141878 (PMC4641725; doi:10.1371/journal.pone.0141878)
Supplement: S1 Appendix — Common questions and answers around the issue of housing pregnant pigs, provided to all participants, as a source of additional information via a hyperlink. (PDF) [file pone.0141878.s001.pdf]

Q1: **What is the average gestation period of a sow and how many piglets are born in each litter?**
A1: A sow is pregnant for "three months, three weeks, and three days", or 115 days total. On average she gives birth to 10-12 piglets in each litter.
Q2: **What is the average life span of a sow in an intensive system?**
A2: On average, sows are culled (slaughtered) by their third or fourth parity (pregnancy).
Q3: **When and why did farmers begin to use gestation stalls as a method of housing for pregnant pigs?**
A3: Farmers began using gestation stalls in the 1950's as a means of addressing problems of food competition and to control the feed intake of individual pigs. By feeding pigs individually, competition between animals was eliminated.
Q4: **What is ESF?**
A4: ESF stands for Electronic Sow Feeder and it is a method of feeding used in group systems that allows farmers the greatest control in individually feeding sows. In a group pen, there are one or more electronic sow feeding stations that pigs enter into one at a time to access a daily amount of food. Each pig wears a tag in one ear that is read by a scanner in the feeder. The feeder then dispenses a ration of food specific to the individuals' weight and dietary needs.
Q5: **How are pigs in groups fed individually?**
A5: Pregnant pigs (sows) need different amounts of food, depending on their weight. When fed in groups, competition between animals can result in some sows eating too much and others going hungry. However, there are other ways to deliver food individually to group-housed pigs. One such high-tech method is known as ESF (Electronic sow feeder). Not all producers find the ESF method suitable for their barns, either because of the cost of setting up and training staff to use ESF, or because of its technical complexity.
Q6: **How much of a sow's life is spent in a gestation stall?**
A6: It is common to keep sows in gestation stalls for the first 105-110 days of pregnancy, at which point they are moved to farrowing crates (similar in size to gestation stalls but differing in design to accomodate piglets) to give birth to their young, where they remain for approximately 28 days. After this time, sows are returned to stalls to be artificially inseminated.
Q7: **What is the size of an average gestation stall?**
A7: Individual stalls are made of metal bars with concrete flooring. They are approximately 26-28 inches wide by 7 feet long.
Q8: **Can sows turn around in stalls?**
A8: Individual stalls allow for sows to stand up, sit and lie down and take about one step forward or backward; however, they do not allow for sows to turn around.
Q9: **What environment and behaviours are natural for pigs?**
A9: Watch a video from Q-Pork Chains Open Learning Platform on a study of pigs in semi-natural environments to learn about natural behaviours and environments for pigs.
Q10: **What are natural behaviours for sows?**
A10: Sows have inherited much of their natural behaviours from their ancestor, the wild boar. In natural, free-ranging environments, 2-3 sows, typically sisters, stay together in a group with their young, until the offspring are older and start their own groups. During the day these social animals graze together, seaching for edible plants, or rooting in the soil for insects, worms and fungi. At night they sleep together in a communal nest. Before giving birth (also known as "farrowing"), sows are highly motivated to build a nest, isolating themselves from the group for the period of farrowing and for the first few days of lactation. Sows wallow in mud or water on hot days to keep themselves cool, as these animals are unable to perspire. Grooming behaviours are also important for these animals. In natural systems, aggression is rare.
Q11: **What kinds of unnatural behaviours occur in both housing systems?**
A11: Sows and gilts in gestation stalls may stand inactive for prolonged periods of time. This may be a response to experiencing difficulty when attempting to lie down, due to the confines of the gestation stall. Sows and gilts may also be observed attempting to root on concrete or metal slatted floors in gestation stalls. Rooting is a behaviour sows and gilts are motivated to perform in natural environments, or when provided with straw or other types of bedding. Nest building before giving birth is a natural behaviour performed by pregnant sows and gilts. When there is no bedding or other materials to manipulate, frustration is evident in sows and gilts and can be reflected in increased postural changes (switching from lying to sitting to standing position), which can also cause skin damage and lesions.
Q12: **What are the typical sow and gilt injuries and health concerns associated with group housing?**

A12: In systems that group house sows, aggressive interactions can occur between unfamiliar sows when they are mixed together to form new groups and when they must be in close proximity to one another due to space restrictions. Wounds on the necks and shoulders of sows are reflective of aggressive attacks due to sows negotiating social rank. Sows that are less dominant may exhibit signs of stress in groups. If feed is distributed all at once in group systems, less dominant sows may have access to less feed, which can result in wounds to hindquarters and can affect weight gain. Some group housing systems use electronic sow feeders (ESF) to individually feed sows. Sows enter a feeding stall with a gate that closes behind them. Once inside, a computer scans their ear tag and dispenses food rations specific to their needs. When sows are entering ESF stalls, other sows, frustrated at not being fed at the same time, have the opportunity to bite the vulva of the sow in the stalls. Lameness (problems walking associated with hoof and leg injuries) is also a concern for sows living in groups on concrete or slatted floors, compared to those that have straw or other types of bedding material covering floor space. Sows that have lived in groups for the duration of their pregnancy have been found to have higher levels of stress, indicated by decreased productivity (number of piglets alive at the end of weaning) and increased skin lesions, when they are moved to farrowing crates where they give birth to and nurse their young for up to a month, compared to sows that lived in gestation stalls for the duration of their pregnancy.

Q13: **What are the typical sow and gilt injuries and health concerns associated with individually housing sows in stalls?**

A13: Sows living in gestation stalls have a decreased ability to exercise control over their environment. This means, for example, that they are unable to respond to changes in environmental temperature, as they would if they were living in semi-natural or natural conditions where they would seek mud, or shade on warm days and would huddle with other pigs to retain body heat on cold days. Additionally, sows cannot avoid aggressive neighbouring sows when they are confined in crates and this can affect stress levels; however, injury rates have been found to be less in gestation stalls compared to groups. Due to the restriction in movement that stalls cause, sows in these systems have been found to have skin lesions from repeated postural changes and have decreased muscle mass, as well as decreased bone and joint strength. Lameness (problems walking associated with hoof and leg injuries) is also a concern for sows living in groups on concrete or slatted floors, compared to those that have straw or other types of bedding material covering floor space.

Q14: **What is the current percentage of sows housed in each system?**

A14: In the U.S. in 2003, over 82% of the total crop from pigs was produced by large farms with sow herd numbers over 5000. In Canada today, there are approximately 1.3 million sows and gilts. It is believed that the majority of these animals spend the term of their pregnancies in gestation stalls.

Q15: **What are the laws in other countries regarding gestation stalls?**

A15: In the European Union legislation has been passed calling for a ban on gestation stall housing (except for the first four weeks of pregnancy) by 2013.

In the U.S., legislation was passed in Florida that banned gestation stalls by 2008 and in Maine by January, 2011. Arizona and Michigan will eliminate their use of stalls by 2012 and in Oregon legislation will take effect in 2013. By 2015, California pig farmers will no longer be able to use gestation stalls. Industry in Colorado decided upon a voluntary phase-out of gestation stalls by 2017.

New Zealand will phase out its use of gestation stalls by 2015 and the pork industry in Australia has announced that they will stop using stalls by 2017.
